# Mechanical axial instability of segmental pedicle screw instrumentation for adolescent idiopathic scoliosis: a retrospective cohort study of tulip screw versus dual locking cup instrumentation

**DOI:** 10.2340/17453674.2025.44038

**Published:** 2025-07-18

**Authors:** Aron FRANTZÉN, Antti SAARINEN, Eetu SUOMINEN, Matti AHONEN, Ilkka HELENIUS

**Affiliations:** 1 Department of Orthopaedics and Traumatology, University of Helsinki and Helsinki University Hospital, Helsinki; 2 Department of Paediatric Surgery, Orthopaedics and Traumatology, University of Turku and Turku University Hospital, Turku; 3 Department of Orthopaedics and Traumatology, University of Turku and Turku University Hospital, Turku; 4 Department of Paediatric Surgery, Orthopaedics and Traumatology, New Children’s Hospital, Helsinki University Hospital and University of Helsinki, Helsinki, Finland

## Abstract

**Background and purpose:**

The effects of axial instability in the rod–screw interface resulting in axial slippage between screws and rods are largely unknown. We aimed to assess the incidence of axial slip and loss of correction by comparing tulip screw versus dual locking cup in spinal instrumentations of patients treated with posterior spinal fusion for adolescent idiopathic scoliosis (AIS). We also aimed to assess whether axial slip would affect health-related quality of life.

**Methods:**

This study consists of 194 patients who underwent posterior spinal fusion for AIS during 2012–2022. All patients had a minimum of 2 years’ follow-up. There were 98 patients treated with segmental tulip pedicle screw instrumentation and 96 patients with segmental dual locking cup constructs. Axial slip was defined as ≥ 2 mm and was assessed by measuring the rod exceeding the last pedicle screw and the distance between the 2 lowest screws on the same rod. Loss of correction was assessed by comparing postoperative and 2-year radiographic measurements. Health-related quality of life was assessed using the SRS-24 questionnaire.

**Results:**

Axial slip occurred only between the lowest instrumented vertebra and the vertebra above it on the convex side of the deformity. At 2 years of follow-up, axial slippage of 2 mm or more was observed more often in the dual locking group, which was observed in 24 (25%) patients in the dual locking cup group and 11 (11%) patients in the tulip group (risk ratio [RR] 2.2, 95% confidence interval [CI] 1.2–4.4). Minimum of 10° loss of major curve correction was found in 1 (1%) patient in the tulip group and 9 (9%) patients in the dual locking group (RR 9.1, CI 1.2–100).

**Conclusion:**

Axial slip was significantly less frequent in the tulip group than in the dual locking cup group. This suggests that tulip screw instrumentation may offer superior mechanical stability in posterior spinal fusion for AIS. Axial slip was not associated with health-related quality of life outcomes.

To prevent further progression of the deformity, adolescent idiopathic scoliosis (AIS) over 45°, according to Cobb [1], usually requires surgical treatment [2]. Posterior spinal fusion with pedicle screw instrumentation with contoured bilateral rods is considered to be the gold standard, as it allows restoration of the spinal balance in both coronal and sagittal planes [3]. Rods are secured to the pedicle screws, leading to a biomechanically stable single construct. Asymmetrical rods are sometimes used to increase the stiffness of the instrumentation [4]. Asymmetrical rods with larger sagittal diameters in comparison with traditional circular rods have been shown to improve thoracic kyphosis restoration [4,5].

Loss of correction after spinal fusion for AIS has been reported for various reasons [6,7]. Skeletal immaturity is associated with loss of correction and adding-on [8]. Continuing anterior column growth after posterior-only fusion may lead to the so-called crankshaft phenomenon [8]. Use of hybrid instrumentation or removal of instrumentation may lead to loss of correction [7,9]. Due to the considerable forces applied to the instrumentation before definitive bone healing is achieved, mechanical instability can cause the rods to experience axial slippage, potentially resulting in a partial loss of correction. Tulip screws lock the longitudinal rods into screw heads by a set screw while dual locking cups are locked without additional set screws. In dual locking cup instrumentation, final locking is achieved by lifting the outer locking cup over the inner rod-bearing cup (Figure 1). A recently published retrospective study with 1 year of follow-up reported axial slip in 27% of patients treated for AIS with non-segmental dual locking cup instrumentation [10].

**Figure 1:**
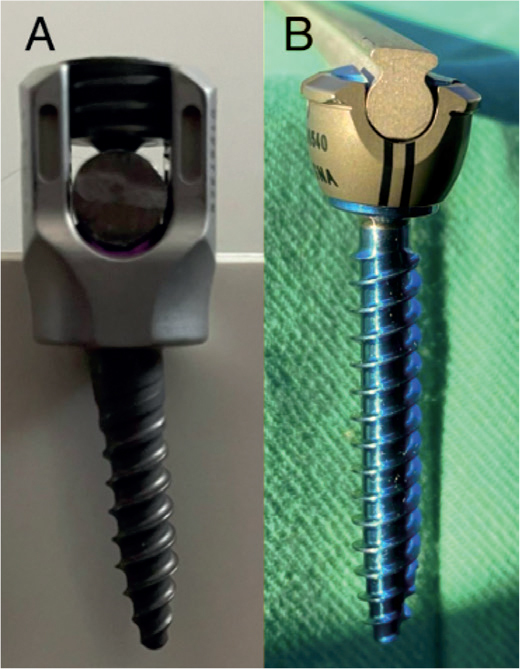
(A) Tulip screw with set screw inserted to secure the rod. (B) Dual locking cup screw with outer locking cup lifted over inner cup to secure the rod.

We aimed to investigate whether axial slip occurs in segmental pedicle screw instrumentation using tulip screws vs dual locking cup screws and to determine whether the extent of axial slip increases over a 2-year follow-up period and causes loss of correction in patients who are undergoing spinal fusion for adolescent idiopathic scoliosis. Furthermore, we aimed to assess whether axial slip would affect health-related quality of life.

## Methods

### Study design

In this retrospective cohort study, 2 different instrumentations (6.0 CoCr Apex Rod, Solera 5.5/6.0 [Medtronic, Minneapolis, MN, USA] with tulip pedicle screws, or Mesa 2 Spinal Deformity [Stryker, Portage, MI, USA] with dual locking cup pedicle screws) were used in the 2 study institutions between 2012 and 2022. Intrumentation used at the 2 institutions was based on surgeon preference. All patients were followed using a standardized protocol before and immediately after the initial surgery and at outpatient clinic controls at 6 months and 2 years after the surgery. All patients were followed for a minimum of 2 years. Implant density is defined as the average number of fixation points placed in each fused level, with implant densities of 1.8 and over defined as high-density constructs [11]. Pedicle screw density was at least 1.7 screws per vertebra for all patients. The study was reported in accordance with the STROBE guidelines.

### Surgical technique

**Figure 2:**
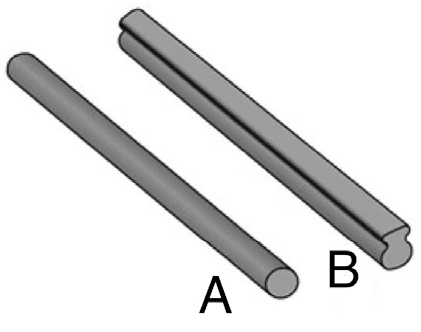
(A) Upper round part of a transition rod. (B) Lower rail rod part of a transition rod. Like rail rods, sagittally reinforced rods have a larger sagittal diameter than round rods but with an oval transverse area.

All patients were operated on by 2 experienced senior pediatric orthopedic surgeons using a standardized technique and high-density pedicle screw constructs. Both surgeons used both tulip and dual locking cup instrumentation. The posterior elements were exposed subperiosteally to the tips of the transverse processes at all segments including the lamina of the lowest instrumented vertebra to facilitate bony healing. Bilateral segmental pedicle screw instrumentation with direct translation and en bloc direct vertebral derotation was performed. Pedicle screws were inserted using free-hand technique. Pedicle screw placement was standardized. 2 to 3 pairs of polyaxial screws were used at the upper end of the construct, with sagittal adjusted (Solera) or uniplanar screws (Mesa) at the mid part of the instrumentation, and the polyaxial screws at the end of the construct (thoracic) and in the lumbar spine. If required, Smith-Petersen osteotomies were performed. Intraoperative computed tomography scans were used to verify pedicle screw positioning [12]. Sagittally reinforced 6 mm cobalt chromium rods (Solera 5.5/6.0) were used for all patients in the tulip screw group and 5.5 mm cobalt chromium transition rods (upper part round for the 2–3 most proximal pairs of the screws and the lower part beam-like rod, MESA2) for all patients in the dual locking cup group (Figure 2). Posterolateral spinal elements were decorticated using a high-speed drill. Local bone from facetectomies and osteotomies with bone graft extender were applied on this bleeding bony surface. Fusion levels were selected using Lenke classification [13]. The last substantially touched vertebra was used as the lowest instrumented vertebra (LIV) in Lenke 1 and 2 curves [14] and L3 or L4 in Lenke 3–6 curves.

### Radiographic parameters

Radiographs were evaluated by 2 independent observers. Standing posteroanterior and lateral radiographs were assessed preoperatively, after the initial surgery, and approximately 6 months and 2 years after the surgery. Proximal thoracic, main thoracic, and thoracolumbar/lumbar curves were measured using the Cobb technique [1]. Thoracic kyphosis (T5–T12) and lumbar lordosis (T12–S1) were measured from the lateral radiographs [15]. Coronal and sagittal alignment were measured. The coronal tilt of lowest instrumented vertebra and L5 vertebra was measured.

***Axial slip.*** To evaluate axial slip the length of the rod exceeding the last pedicle screws and the distance between the pedicle screws on the lowest instrumented vertebra (LIV) and LIV–1 were measured. In case of rod slipping during the follow-up (axial slip), a senior orthopedic spine surgeon measured the rods and verified the slip. Axial slip of 2 mm or more was defined as an indicator of mechanical instability of the construct. Rod diameter was used in calibration of measurements.

***Loss of correction.*** Loss of correction was assessed by comparing postoperative and 2-year radiographic measurements (major curve, kyphosis, lordosis, change in coronal balance, change in sagittal balance).

### Health-related quality of life

A scientifically non-validated Finnish translation of the condition-specific Scoliosis Society Score 24 (SRS-24) questionnaire was used to assess the health-related quality of life of the patients [16]. Health-related quality of life was assessed preoperatively and at 2 years. Higher score indicates better patient-reported outcomes. The SRS-24 questionnaire consists of 7 domains: pain, function, self-image, activity, postoperative self-image, postoperative function, and satisfaction.

### Statistics

All continuous data are presented as mean with standard deviation (SD) and range or with median together with lower and upper quartiles (IQR). Categorical variables are reported as counts with percentages. Risk ratios (RRs) with 95% confidence intervals (CI) were calculated to express the risk of mechanical instability parameters between the 2 cohorts. Adjusted risk ratios with CIs were calculated for axial slip using Poisson regression with robust standard errors using age and sex as covariates.

Univariable logistic regression was performed to identify potential risk factors for ≥ 10° loss of main curve correction. Interobserver and intraobserver reliability were calculated for the length of the rod exceeding the last pedicle screws using Cohen’s kappa. A kappa value of 0.41–0.60 indicates moderate agreement, 0.61–0.80 indicates substantial agreement, and 0.81–1.00 indicates almost perfect agreement [17]. Intraobserver reliability for the measurements was 0.75, indicating a substantial level of agreement between the measurements. Interobserver reliability for the measurements was 0.70 between the 2 independent observers, indicating a substantial level of agreement between the measurements.

Comparison of SRS data between groups was performed using a Wilcoxon rank sum test. All tests were performed as 2-sided with a significance level set at 0.05. Statistical analysis was performed using R 4.4.2 (R Foundation for Statistical Computing, Vienna, Austria).

### Ethics, registration, data sharing plan, funding, and disclosures

The study has ethical committee approval and informed consent to participate was obtained from all participants (ETMK 38/1800/2015). Data can be shared upon reasonable request. This study was funded by Finnish State Funding via Helsinki and Turku University Hospitals, and Grants to Institution from the Finnish Paediatric Research Foundation, Liv och Hälsa Foundation, Päivikki and Sakari Sohlberg Foundation, Finska Läkaresällskapet, Medtronic, and Stryker. The funding bodies were used for salaries of the researchers, research nurses, and biostatistician and they had no role in the interpretation, analyses, or writing of this manuscript. The funding body from Stryker was used solely for the analyses of the dual locking cup instrumentation group. Medtronic has supported this study financially through the External Research Program (ERP-2024-13925), and was not involved in the study design, collection, analysis, and interpretation of the data. Complete disclosure of interest forms according to ICMJE are available on the article page, doi: 10.2340/17453674.2025.44038

## Results

**Figure 3:**
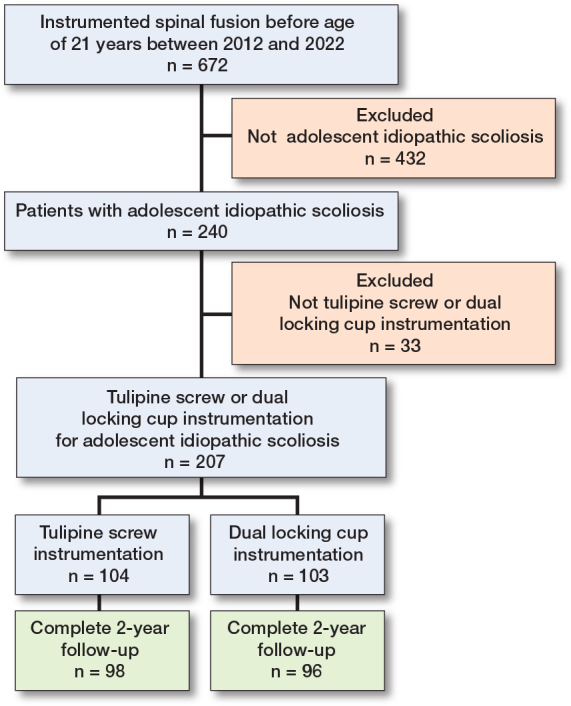
Flowchart.

672 patients had undergone instrumented spinal fusion before age 21 at the 2 study institutions between 2012 and 2022. Of these patients, 207 were operated on for AIS using tulip screw or dual locking cup instrumentation. 98 patients in the tulip group and 96 patients in the dual locking cup group had complete 2-year follow-up and were included in the study (Figure 3, Table 1). The mean age for the tulip group was 15.8 (SD 1.9) years and 15.6 (SD 2.2) years for the dual locking cup group. Preoperative characteristics were similar between the groups.

**Table 1:** Clinical characteristics of the study groups. Data presented in means with standard deviation (SD) and number of subjects

Item	Tulip screw	Dual locking cup
	(n = 98)	(n = 96)
Age at surgery, mean (SD)	15.8 (1.9)	15.6 (2.2)
Lenke classification, n		
I	36	29
II	34	33
III	4	11
IV	8	3
V	3	13
VI	13	7
Number of levels fused, mean (SD)	11 (2)	11 (3)
Posterior column osteotomies, mean (SD)	0.85 (1.4)	0.42 (0.97)

Major curve correction was significantly better in the tulip than in the dual locking cup instrumentation group (mean difference –11.8°, CI –15.3 to –8.3, Table 2). Thoracic kyphosis restoration was significantly better in the dual locking cup than in the tulip instrumentation group during the follow-up (mean difference –11.0°, CI –14.3 to –7.7, Table 2). There were significantly more posterior spinal osteotomies performed in the tulip group than in the dual locking cup group (P = 0.002).

**Table 2:** Radiographic characteristics of the study groups presented in means with standard deviations (SD)

Characteristics	Tulip	Dual	Mean
	screw	locking cup	
	mean (SD)	mean (SD)	difference (CI)
Cobb angle (°)			
Preoperative	52 (8.1)	52 (7.6)	–0.5 (–2.8 to 1.7)
Postoperative	12 (4.0)	16 (6.6)	–3.6 (–5.1 to –2.0)
6 months	11 (5.6)	17 (7.1)	–6.0 (–7.9 to –4.1)
2 years	12 (6.1)	18 (7.4)	–6.3 (–8.3 to –4.3)
Curve correction (%)	77 (11)	65 (13)	–11.8 (–15.3 to –8.3)
Thoracic kyphosis (T5–T12, °)			
Preoperative	21 (12)	23 (14)	–2.5 (–7.6 to 2.7)
Postoperative	18 (6.4)	28 (13)	–10.4 (–13.2 to –7.5)
6 months	19 (6.9)	30 (14)	–11.7 (–15.0 to –8.3)
2 years	19 (7.1)	30 (14)	–11.0 (–14.3 to –7.7)
Lumbar lordosis (T12–S1, °)			
Preoperative	51 (12)	57 (15)	–5.8 (–11.2 to –0.3)
Postoperative	47 (10)	49 (14)	–1.5 (–4.9 to 2.0)
6 months	50 (12)	53 (12)	–3.3 (–6.8 to 0.2)
2 years	51 (12)	54 (13)	–2.8 (–6.5 to 1.0)
Coronal balance (C7–central sacral vertical line, mm)			
Preoperative	16 (13)	13 (13)	3.2 (–1.6 to 7.9)
Postoperative	14 (11)	–1.4 (19)	15.3 (10.9 to 19.7)
6 months	11 (8.6)	–1.8 (16)	12.8 (9.0 to 16.6)
2 years	9.8 (8.2)	–0.9 (16)	10.7 (6.9 to 14.4)
Sagittal balance (C7–S1, mm)			
Preoperative	15.1 (23)	9.8 (33)	5.3 (–5.3 to 15.8)
Postoperative	13 (26)	14 (24)	–1.2 (–8.4 to 6.1)
6 months	5.8 (23)	8.3 (23)	–2.5 (–9.4 to 4.3)
2 years	0.8 (25)	3.9 (24)	–3.1 (–10.4 to 4.2)

CI = 95% confidence interval.

1 patient was reoperated on for screw malposition in the tulip screw group, which resulted in a transient postoperative neurologic deficit. Additionally, 2 patients in this group presented with delayed cerebrospinal fluid leakage and postural headache requiring screw revision. In the dual locking cup group, 1 patient required reoperation for deep surgical site infection and 1 patient for screw malposition.

### Axial slip and loss of initial radiographic correction

**Figure 4:**
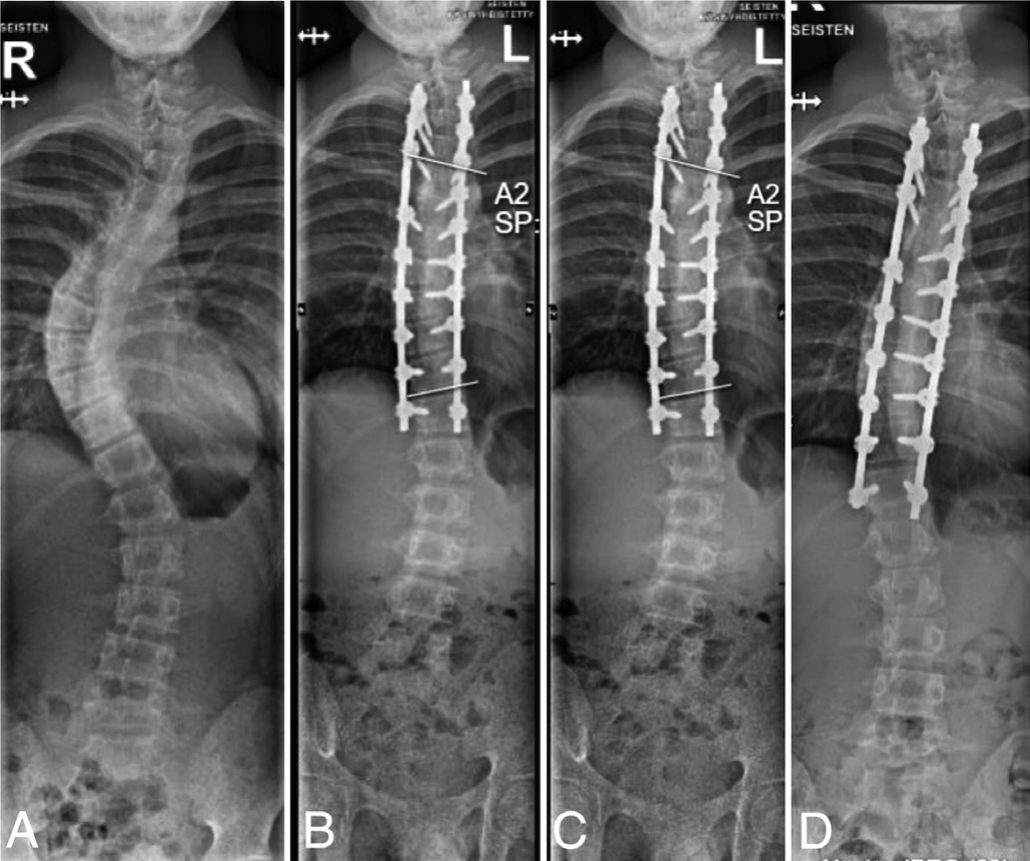
(A) 14-year-old girl with 54° isolated right thoracic adolescent idiopathic scoliosis (Lenke 1BN). (B) Dual locking cup instrumentation from T3 to T12 using cobalt chromium transition range-rail rods. Gradually increasing axial slip > 5 mm between T11 and LIV resulting in coronal malalignment can be observed on the (C) 6-month and (D) 2-year radiographs. (D) Note the shortening of the residual rod below the right T12 pedicle screw.

**Figure 5:**
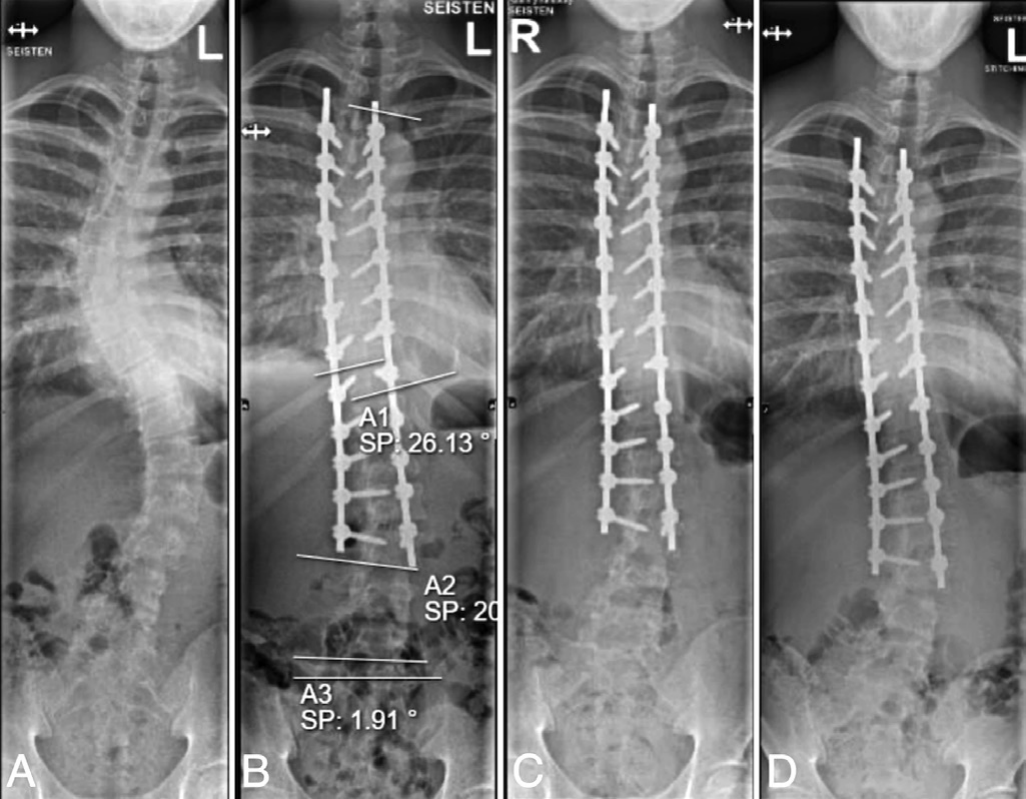
(A) 15-year-old boy with 51° left thoracolumbar adolescent idiopathic scoliosis (Lenke 6CN). (B) Dual locking cup instrumentation from T4 to L3 using cobalt chromium transition range-rail rods. Note the axial slip > 5 mm between L2 and LIV resulting in loss of lumbar curve correction on the (C) 6-month and (D) 2-year radiographs. (D) Progressive shortening of the residual rod below the left L3 pedicle screw can be observed.

**Figure 6:**
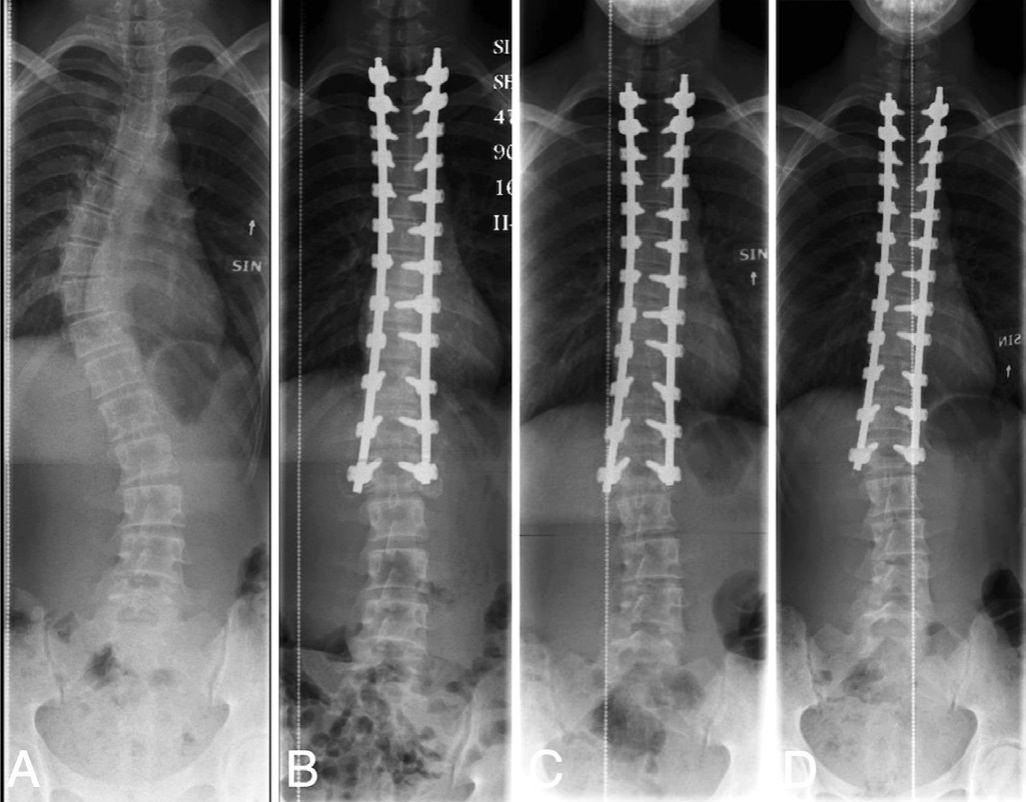
(A) 15-year-old girl with 52° right thoracic adolescent idiopathic scoliosis (Lenke 2AN). (B) Instrumentation from T2 to L2 using tulip screws with sagittal reinforced cobalt chromium rods. Axial slip of 2 mm between the L1 and LIV on the (D) 2-year radiograph, which showed minimal increase during follow-up and resulted in minor loss of major curve correction and coronal malalignment in comparison with (B) postoperative and (C) 6-month radiographs.

Axial slip of 2 mm or more at 2-year follow-up was observed in 11 (11%) and 24 (25%) patients of the tulip and dual locking cup groups respectively (RR 2.2, CI 1.2–4.4, Table 3). Axial slip of 5 mm or more was observed in 4 (4%) and 7 (7%) patients of these groups respectively (RR 1.8, CI 0.54–5.9). Axial slip occurred solely between the LIV and the vertebra above it (Figures 4–6). Axial slip occurred only on the convex side of the deformity. This slip tended to increase during the 2-year follow-up in patients experiencing this phenomenon and it was observed both in thoracic and thoracolumbar spinal fusions (Figures 4–6). Adjusted risk ratio analysis showed that the risk of axial slip ≥ 2 mm was lower in the tulip screw group compared with the dual locking cup group (RR 0.45, CI 0.23–0.87). Male sex was associated with an increased risk (RR 2.1, CI 1.1–3.6), while age was not significantly associated with the outcome. For axial slip ≥ 5 mm, no significant difference was observed between the instrumentation groups (RR 0.56, CI 0.17–1.85), although male sex remained a significant risk factor (RR 5.5, CI 1.7–17.7). Loss of major curve correction of 10°or more was found in 1 (1%) of the tulip and 9 (9%) of the dual locking cup groups (RR 9.1, CI 1.2–100).

**Table 3:** Radiographic characteristics of the study groups with loss of initial radiographic correction at 2 years. Axial slip was evaluated by measuring rod exceeding the most caudal screw and distance between 2 lowest screws on same rod

Characteristics	Tulip	Dual locking	Risk ratio (CI)
	screw, n	cup, n	
Axial slip			
≥ 2 mm	11	24	0.45 (0.23–0.87)
≥ 5 mm	4	7	0.56 (0.17–1.85)
Loss of major curve correction			
≥ 10°	1	9	0.11 (0.01–0.84)
Loss of major thoracic curve correction			
≥ 10°	1	4	0.24 (0.03–2.15)
Loss of thoracolumbar curve correction			
≥ 10°	9	6	1.47 (0.54–3.97)
Change in coronal balance			
≥ 10 mm	12	19	0.62 (0.32–1.20)
≥ 20 mm	1	10	0.10 (0.01–0.75)
Change in sagittal balance			
≥ 10 mm	19	29	0.64 (0.39–1.06)
≥ 20 mm	10	14	0.70 (0.33–1.50)

CI = 95% confidence interval.

Change in the coronal balance (≥ 20 mm, postoperative vs 2-year follow-up) was observed in 1 (1%) and 10 (10%) of the tulip and dual locking cup groups (RR 10, CI 1.3–100). Axial slip did not affect the sagittal balance. At 2-year follow-up, the mean change in the distance between the 2 lowest screws on the right side was +0.8 mm in the tulip group and –0.3 mm in the dual locking cup group, with a mean difference of –1.2 mm (CI –1.8 to –0.6), and on the left side +1.2 mm and +0.3 mm, respectively, with a mean difference of –0.9 mm (CI –1.6 to –0.30).

### Risk factors for loss of radiographic curve correction

There were no significant correlations between preoperative curve characteristics and loss of coronal correction or in the incidence of axial slip in either of the groups.

### Health-related quality of life outcomes

SRS-24 total and domain scores are presented in Table 4. Health-related quality of life was not associated with axial slip in the tulip or dual locking cup groups. There was no difference in health-related quality of life between the tulip and dual locking cup groups.

**Table 4:** SRS-24 Outcome Questionnaire Domains in the study groups. Data presented in means with standard deviation. The highest possible value is 5.0 and a higher value reflects better outcome

Item	Dual locking cup			Tulip screw		
	No slip	Slip	P value	No slip	Slip	P value
Preoperative						
Total	4.2 (0.87)	4.3 (0.37)	1	4.1 (0.73)	4.1 (0.47)	0.7
Pain	3.8 (0.86)	4.0 (0.85)	0.3	3.4 (1.00)	3.6 (0.39)	0.6
Function	4.3 (0.33)	4.3 (0.33)	0.9	4.3 (0.67)	4.3 (0.00)	0.2
Self-image	4.0 (1.33)	3.3 (1.50)	0.3	4.0 (1.00)	3.7 (0.92)	0.7
Activity	4.7 (0.67)	5.0 (0.67)	0.6	4.7 (1.08)	4.8 (1.17)	0.9
2 years						
Total	4.1 (0.58)	4.4 (0.54)	0.2	4.1 (0.60)	4.1 (0.13)	0.8
Pain	4.4 (0.71)	4.9 (0.64)	0.07	4.4 (1.00)	4.6 (0.43)	0.9
Function	4.3 (0.00)	4.3 (0.00)	0.4	4.3 (0.33)	4.3 (0.00)	0.6
Self-image	4.3 (1.00)	4.3 (0.83)	0.7	4.0 (0.83)	4.0 (0.67)	0.6
Activity	4.7 (0.33)	5.0 (0.17)	0.05	5.0 (0.33)	5.0 (0.00)	0.2
Postoperative						
self-image	3.0 (0.67)	3.7 (1.00)	0.3	3.0 (0.67)	3.0 (0.33)	0.6
function	3.0 (0.00)	3.0 (0.00)	0.5	3.0 (0.75)	3.0 (1.00)	0.2
Satisfaction	4.3 (0.67)	4.7 (0.67)	0.2	4.3 (1.00)	4.3 (0.33)	0.8

## Discussion

We aimed to investigate whether axial slip, and loss of correction in relation to axial slip, occurs in segmental pedicle screw instrumentation using tulip screw vs dual locking cup screw instrumentation. In addition, we also wanted to see whether axial slip affects health-related quality of life during the follow-up. We showed that axial slip and loss of correction occurred in both instrumentation types, although significantly more in the dual locking cup group. Health-related quality of life was not affected by axial slip.

Axial slippage was observed significantly more often in the dual locking cup system (25%) than in the tulip system (11%). This resulted in significantly lower major curve correction (77% vs 65%) and increased risk of coronal imbalance (1% vs 10%) in the dual locking cup group at 2-year follow-up. Improved curve correction in the tulip group could, however, be influenced by the significantly higher number of osteotomies performed [18]. Male sex was associated with an increased risk of axial slip while age was not. A multicenter study from 2007 reported similar loss of correction between males and females treated with spinal fusion for AIS [19]. Age has previously been associated with loss of correction [6,7]. Thoracic kyphosis restoration was significantly better in the dual locking group.

Our findings confirm the previously reported finding of axial slip when using dual locking cup instrumentation with round rods for AIS [10]. However, a similar finding was also observed for tulip screw instrumentation, but with a much smaller risk. Additionally, the axial slip tended to increase from 6 months to 2-year follow-up and axial slip occurred despite using segmental pedicle screw instrumentation.

Asymmetric rods have been shown to enhance sagittal malalignment restoration in adolescent idiopathic scoliosis [4,5,20]. Although a special high stiffness rod configuration can be used, it might not be an advisable choice if stability between screws and rods is jeopardized as described by the current study and by Schlösser et al. 2024 [10]. Mishandling instrumentation by, e.g., forcefully engaging rods into tulips could also decrease axial stability. In our study, both instrumentations showed evidence of this phenomenon, but it was significantly more pronounced in the dual locking cup constructs and resulted in both loss of correction in the coronal plane and coronal imbalance in patients operated on using dual locking cup instrumentation. Axial slip tended to increase during follow-up and was observed in both the lower thoracic and lumbar LIVs. However, axial mechanical instability did not result in inferior health-related quality of life outcomes or revision surgery. The authors could not identify further risk factors for mechanical instability, e.g., thoracic vs lumbar instrumentation or smaller vs larger initial curve size. Based on the findings of our study, the caudal end of the construct should include tulip screws in patients with dual locking cup instrumentation. The risk of axial slip in patients with tulip instrumentation was significantly lower and thus no changes in our clinical practice have been planned.

### Limitations

A retrospective comparative study of prospectively collected data on a consecutive patient group operated on using 2 different instrumentation systems was performed. Study groups of equal and relatively large size were compared. Independent observers measured radiographic outcomes. Intra- and inter-observer repeatability was substantial.

Cobalt chromium rods were used, but cross-links were not used. An axial slip of 2 mm or more was used as an indicator of axial mechanical instability. Whether this small radiographic finding is significant in terms of, e.g., increased risk of non-union in the long-term follow-up remains unclear. Similarly, whether a minimum of 3 months’ activity restriction after instrumented posterior spinal fusion is adequate can be discussed. There were more posterior osteotomies in the tulip pedicle screw group. Results of the current study are limited by a lack of biomechanical testing. Further in vitro studies are needed to validate the findings of this study.

### Conclusion

Axial mechanical instability was observed in 11% of tulip and 25% of dual locking constructs after instrumented posterior spinal fusions for adolescent idiopathic scoliosis. Both loss of major curve correction (≥ 10°) and coronal imbalance were significantly more common in the dual locking cup constructs than in the tulip screw constructs. Major curve correction was significantly better in the tulip than in the dual locking cup groups. Axial slip was not associated with inferior health-related quality of life outcomes during the follow up.

*In perspective,* multicenter register-based studies are needed to confirm whether these differences exist in a wider perspective and longer-term follow-up to investigate whether this is associated with increased risk of non-union. Risk of axial slip should be taken into consideration when selecting instrumentation for adolescents needing instrumented spinal fusion for idiopathic scoliosis.

## Notes

AF: Writing—original draft, methodology, gathering and processing of data. AJS: writing—original draft, conceptualization, methodology, statistical analysis. ENS: writing—original draft, methodology, gathering and processing of data. MA: writing—review and editing, methodology. IH: writing—review and editing, conceptualization, supervision. All authors have read and agreed to the published version of the manuscript.

Handling co-editors: Paul Gerdhem and Robin Christensen

*Acta* thanks Acke Ohlin and other anonymous reviewers for help with peer review of this manuscript.
